# Methyltransferase Set7/9 as a Multifaceted Regulator of ROS Response

**DOI:** 10.7150/ijbs.83158

**Published:** 2023-04-23

**Authors:** Alexandra Daks, Oleg Shuvalov, Olga Fedorova, Sergey Parfenyev, Hans-Uwe Simon, Nickolai A. Barlev

**Affiliations:** 1 Institute of Cytology, Russian Academy of Sciences, 194064, St Petersburg, Russian Federation.; 2 Institute of Fundamental Medicine and Biology, Kazan Federal University, 420008, Kazan, Russian Federation.; 3 Institute of Pharmacology, University of Bern, 3010, Bern, Switzerland.; 4 School of Medicine, Nazarbayev University, 010000, Astana, Kazakhstan.

**Keywords:** Set7/9, ROS metabolism, oxidative stress, p53, chromatin, diabetes, myocardial ischemic injury, nephropathy

## Abstract

Reactive oxygen species (ROS) induce multiple signaling cascades in the cell and hence play an important role in the regulation of the cell's fate. ROS can cause irreversible damage to DNA and proteins resulting in cell death. Therefore, finely tuned regulatory mechanisms exist in evolutionarily diverse organisms that are aimed at the neutralization of ROS and its consequences with respect to cellular damage. The SET domain-containing lysine methyltransferase Set7/9 (KMT7, SETD7, SET7, SET9) post-translationally modifies several histones and non-histone proteins via monomethylation of the target lysines in a sequence-specific manner. *In cellulo*, the Set7/9-directed covalent modification of its substrates affects gene expression, cell cycle, energy metabolism, apoptosis, ROS, and DNA damage response. However, the *in vivo* role of Set7/9 remains enigmatic. In this review, we summarize the currently available information regarding the role of methyltransferase Set7/9 in the regulation of ROS-inducible molecular cascades in response to oxidative stress. We also highlight the *in vivo* importance of Set7/9 in ROS-related diseases.

## Introduction

Reactive oxygen species (ROS) include the superoxide anion (O^2-^), hydrogen peroxide (H_2_O_2_) and the hydroxyl radical (HO•), which are produced during the normal life cycle of cells 1. Being a by-product of aerobic metabolism, ROS operate as signaling molecules that participate in the regulation of processes such as cell differentiation, energy metabolism, senescence, and immune response 2-5. Due to the high reactivity of ROS, their excessive levels are deleterious to cells and induce different types of lesions of such macromolecules as DNA, RNA, proteins, and lipids, thereby disrupting cellular homeostasis 6, 7. Accordingly, finely tuned regulatory mechanisms exist in evolutionarily diverse organisms that alleviate the consequences of ROS-inflicted damage.

As mentioned above, ROS can be generated as a result of endogenous cellular processes such as an electron flux from the mitochondrial transport chain, byproducts of enzymatic activity of NADPH oxidases, etc. Furthermore, the level of intracellular ROS may be augmented by external stimuli such as high glucose levels, exposure to hyperoxide ions, UV irradiation, or an increased concentration of metal ions 8-10.

On the molecular level, a particular form of cellular response to ROS insult may vary and depends on the intensity of oxidative stress. Thus, low and moderate levels of ROS may be effectively neutralized by specific antioxidant and cytoprotective enzymes. For example, superoxide anion (O^2-^) is reduced by SOD enzymes (MnSOD, Cu/ZnSOD, and extracellular SOD) to H_2_O, while catalase (CAT) and glutathione peroxidase (GPX) further neutralize hydrogen peroxide (H_2_O_2_) with the formation of water molecules and free oxygen 11. In addition, a moderately augmented level of ROS induces the expression of genes coding for these antioxidant enzymes and redox-regulated chaperones 12. This forms a positive feedback loop mechanism, which eventually results in ROS neutralization and cell survival. Additionally, moderately increased ROS causes cell cycle arrest, and the activation of the DNA repair system 13.

On the contrary, a high level of ROS may overload the capacity of the intracellular glutathione peroxidase detoxification system and hence cause apoptosis induced by lipids peroxidation, mitochondria permeabilization, and the accumulation of fatal DNA damage 14.

It should be noted that at low level ROS stimulate the proliferation of cells. This phenomenon is often observed in cancer cells (especially in RAS mutant cells). In contrast, high doses of ROS mediate deleterious effects on the cells, which, on the other hand, can be attenuated by the glutathione-dependent scavenger system 15, 16.

Both excessive ROS accumulation and the disruption of cellular ROS neutralization are shown to play crucial roles in the development of various diseases and pathological processes. Notably, negative ROS influence has been shown to promote carcinogenesis, including metastasis formation and radio-resistance, as well as chronic inflammatory diseases, Alzheimer's disease, ischemia-reperfusion injury, diabetic heart and vascular disease, insulin resistance, infertility, organ failure, and many others 17-21.

The elevation of ROS levels and dysregulation of redox homeostasis affect numerous intracellular processes and machineries such as gene expression, energy metabolism, molecular signaling, and epigenetic modifications 22. There are several transcription factors (TFs) known to respond to ROS accumulation, including Hif1a, Nrf2, p53, FOXO, NFkB, and AP-1 23-28. These TFs activate the expression of genes whose products participate in ROS neutralization, DNA repair, proteostasis, and cell death, thereby forming an intertwined network of molecular pathways through which ROS affect virtually all cellular processes 29-32.

ROS affecting the activity of a plethora of TFs eventually leads to changes in chromatin modifications and subsequent epigenetic reprogramming 33. Among several epigenetic modifiers that are recruited to chromatin by TFs, the most crucial ones are acetyltransferases and methyltransferases 33, 34.

Numerous reports strongly argue that epigenetic modifiers are not restricted only to histone substrates but can also covalently modify non-histone proteins, including TFs. In line with this is the fact that the protein methylation patterns were found to be altered when subjected to oxidative stress 35, 36. The consequences of lysine methylation can be pleiotropic: from affecting the protein's stability and its cellular localization to altering the interactome with other proteins and nucleic acids (e.g. 37, 38).

The SET domain-containing lysine methyltransferase Set7/9 (aka KMT7, SETD7, SET7, Set9) post-translationally modifies several histones and non-histone proteins by transferring methyl groups onto the target lysines of its substrates. Initially discovered *in vitro* as a histone H3K4-specific methyltransferase, Set7/9 was subsequently shown to fail to efficiently methylate nucleosomal histones, which is a *bone fide* target of true epigenetic modifiers 39. Instead, a number of non-histone targets such as p53 40, estrogen, and androgen receptors (ERα and AR) 41, 42, YAP 43, p65/RelA^44^, PARP1 45, TAF10 46, DNMT1 47, Sam68 48, to name a few, were shown to be methylated by Set7/9 both *in vitro* and *in cellulo* 37, 49 suggesting that Set7/9 likely operates as a factor-specific lysine methyltransferase rather than the histone-specific KMT. Depending on the nature of the substrate protein, Set7/9-directed covalent modification may affect gene expression, cell cycle, energy metabolism, apoptosis, tumor transformation, and DNA damage response (reviewed in 50, 51). Set7/9 was also shown to regulate ROS signaling at different levels and with various, sometimes opposite, effects.

This review highlights the currently underexplored role of methyltransferase Set7/9 in the regulation of ROS signaling and oxidative stress response and subsequently raises a question of whether inhibitors of Set7/9 activity can be used to therapeutically alleviate the consequences of ROS imbalance.

## The effect of Set7/9 on NFkB signaling

In recent years, there has been a lot of information published on the participation of the NFkB signaling pathway in the oxidative stress cell response. Importantly, the role of ROS in the modulation of NFkB activity has also been actively studied. Depending on the cellular and environmental context, ROS stress (especially the one caused by H_2_O_2_) can either activate or inhibit transcriptional functions of NFkB. When activated, NFkB regulates a variety of effector genes including antioxidants, ROS-producing proteins and pro-inflammatory genes (reviewed in 52).

The current paradigm implies that ROS accumulation activates the cytoplasmic IKK kinase complex, which then phosphorylates the IkB complexed with NFkB. The latter anchors the transcriptionally active NFkB subunits p65 (RelA) and p50 in the cytoplasm. Thus, the phosphorylation leads to the dissociation of the IkB-p65/p50 complex, resulting in the proteasomal degradation of IkB and the subsequent translocation of the p65-p50 heterodimer to the nucleus. An IKK-independent phosphorylation of the IkB complex has also been reported, which leads to a similar outcome, i.e. translocation of the released p65-p50 into the nucleus and subsequent activation of the NFkB target genes, a large portion of which are involved in the ROS response 52, 53.

Notably, the ROS-induced NFkB response is multi-faceted since it can activate the expression of both antioxidant proteins, including MnSOD, Cu/ZnSOD, CAT 52, 54-57; and ROS-producing proteins, such as COX2, iNOS, XOR, and others 52, 58, 59. It is well documented that ROS accumulation promotes apoptosis, while ROS neutralization restores normal functioning of the cell. Accordingly, NFkB plays a dual role in cell survival under oxidative stress conditions. For example, after their treatment with H_2_O_2_, HEK293 cells with a knock-down of RelA survived better than those with active RelA 60. On the contrary, H9c2 cardiac myocytes with a silenced expression of RelA demonstrated poor survival in response to H_2_O_2_ 61. A pro-survival effect of NFkB was also observed in Huh7 hepatocarcinoma cells by Qiao et al. 62. There it was shown that the inhibition of NFkB translocation to the nucleus by mutant IkB led to increased apoptosis after the H_2_O_2_ treatment 62. Thus, the role of NFkB in ROS signaling apparently depends on the cellular context and the intensity of oxidative stress.

### Set7/9 promotes the expression of p65/RelA

Several studies published to date suggest that Set7/9 modulates the NFkB signaling pathway through various mechanisms, resulting in different outcomes in respect to the cellular response to ROS. For example, it was shown that high glucose levels promote oxidative stress in the cells 63, 64. In response to glucose-induced oxidative stress in aortic endothelial cells, HAEC, Set7/9 augmented the level of H3K4 methylation at the p65/RelA promoter region. Increased H3K4 methylation correlated with transcriptional activation of the *RELA* gene 65. Subsequently, Set7/9-mediated p65/RelA activation increased the expression of pro-atherogenic NFkB targets VCAM-1 and MCP-1 levels. On the contrary, Set7/9 knock-down prevented the expression of p65/RelA target genes 65.

However, it should be noted that chromatinized histone H3 is a poor substrate for Set7/9 39, 40. Therefore, it is likely that the effect of Set7/9 on NF-kB-mediated transcription was mediated via the methylation of some other non-histone transcriptional factors (see below). Alternatively, Set7/9 may enhance specific H3K4 methylation indirectly, through promoting the recruitment of another histone lysine methyltransferase (KHMT) to the regulatory regions of NFkB-dependent genes. In this respect, it would be interesting to analyze the interactome of Set7/9 in these cells to see whether it can physically bind chromatin modifying enzymes.

Another study points to the role of Set7/9 as a positive regulator of NFkB function. J. Okabe et al. explored the participation of Set7/9 in response to the hyperglycemia of vascular HMEC-1 cells 66. Thereby, it was confirmed that the depletion of Set7/9 led to a reduced expression of crucial pro-inflammatory NFkB targets ICAM-1 and IL-8, while overexpressed Set7/9 caused augmented levels of mentioned NFkB-dependent genes 66.

### Set7/9 directly interacts with p65/RelA and modulates NFkB-dependent transcription

As an alternative to the H3K4-methylation-dependent mechanism that mediates up-regulation of p65/RelA expression, Li et al. showed that Set7/9 can physically interact with the p65/RelA protein and act as a transcriptional co-factor at the promoters of NFkB target genes 67. This research group demonstrated that in HEK293T cells and THP-1 monocytes treated with TNFa the artificially reduced expression of Set7/9 was concomitant with the down-regulation of NFkB-dependent pro-inflammatory genes (such as *CCL2* (MCP-1), *TNF* (TNFa), *CXCL8* (IL-8) etc.). Based on these data, the authors suggested that Set7/9 enhanced the p65/RelA binding to NFkB-regulated promoters and stabilized the p65/RelA complex at these sites 67.

Later, C.-K. Ea and D. Baltimore published the work demonstrating that Set7/9 was able to methylate p65/RelA at K37 in response to stimulation with TNFα 44. This modification was shown to take place both *in vitro* and *in cellulo* using HEK293T and HeLa cells. The authors also revealed the key role of RelA-K37 methylation in the transcription of its target genes. Accordingly, depletion of Set7/9 in the cells attenuated the expression of NFkB-dependent genes, including *NFKBIA* (IkB), *TNF*, and *CXCL10* (IP-10). Interestingly, in U2OS osteosarcoma cells Set7/9 had no effect on p65/RelA methylation at K37 44. In stern contrast to this study, another work suggested that Set7/9 acts as a negative regulator of NFkB transcriptional activity and that Set7/9-mediated methylation of p65/RelA at the K314 and K315 sites was required for this repression 68. Furthermore, it was shown that p65/RelA methylation at K314/K315 promoted its subsequent ubiquitin-dependent proteasomal degradation. Notably, proteasomes themselves can be subjected to ubiquitination in response to different stimuli, thus further complicating this regulatory circuit 69. Accordingly, either the depletion of Set7/9, or mutations of the target lysines into non-methylatable arginines in the p65/RelA protein (K314/315R) was shown to enhance the NFkB-mediated transcription. In particular, cells treated with TNFa displayed activation of NFkB target genes coding for IL-6 and IL-8. Interestingly, the expression of another NFkB-dependent gene, *TNFAIP3* (A20), was not affected by Set7/9 knock-down because, apparently, the regulation of the A20 promoter region was Set7/9 independent 68.

**Figure 1:**
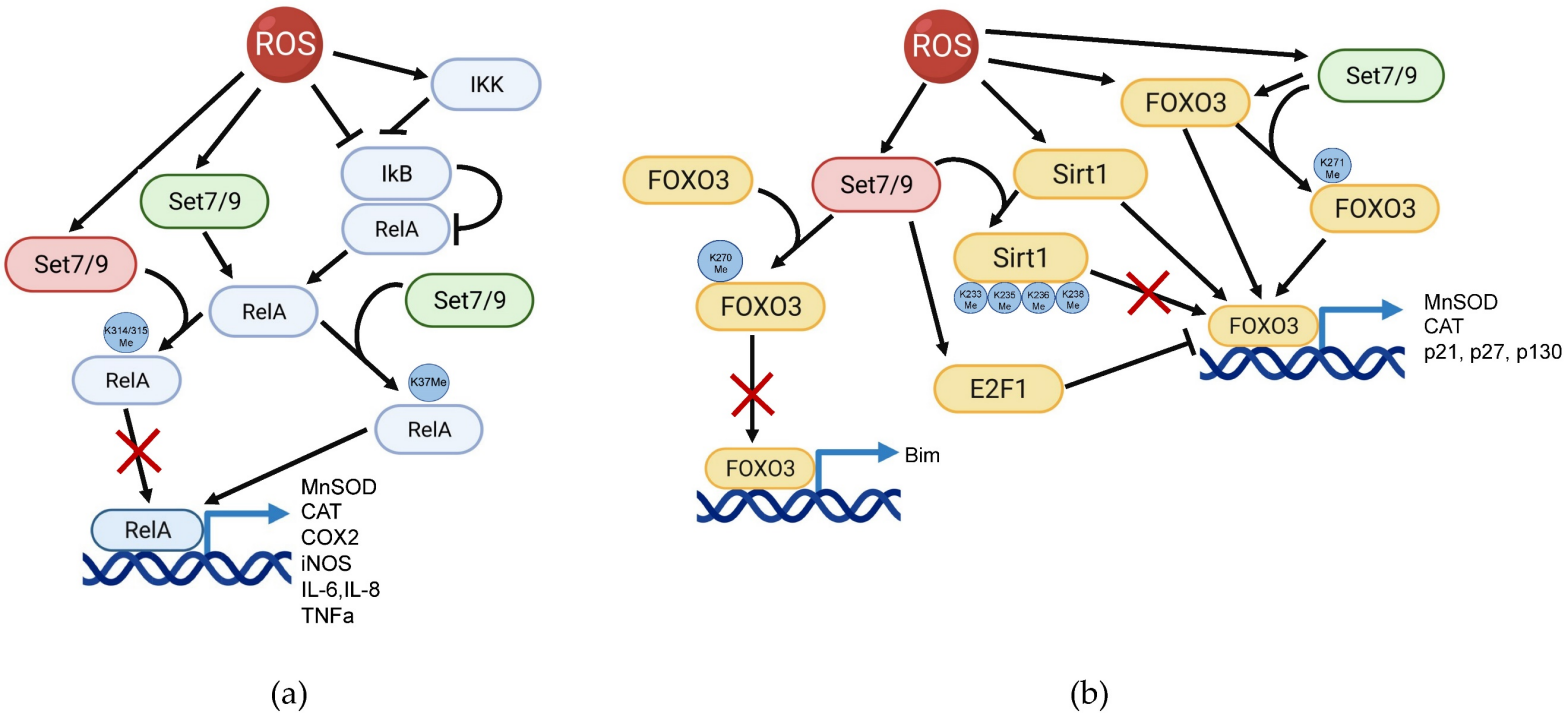
Set7/9 is a regulator of ROS-induced NFkB and FOXO3 signaling pathways. **(a)** Set7/9 may act both as an activator (green) or a suppressor (red) of NFkB in ROS response. Set7/9 augments the expression level of RelA via K37 methylation thereby promoting transactivation of NFkB target genes. On the contrary, Set7/9-mediated methylation of RelA on K314/K315 inhibits its binding to the promoter regions of target genes; **(b)** Set7/9 positively regulates transcription activity of FOXO3 by methylating it on K271. Furthermore, Set7/9-dependent methylation of FOXO3 on K270 prevents the activation of pro-apoptotic Bim. In addition, Set7/9 upregulates the activity of E2F1 that in turn suppresses FOXO3 activity and mediates Sirt1 methylation on lysine residues 233, 235, 236 and 238 resulting in Sirt1 inhibition of its deacetylation activity. The latter prevents Sirt1-mediated activation of FOXO3.

The above-described studies argue that Set7/9-mediated regulation of NFkB is rather complex and apparently depends on the cellular context (Figure 1A).

## The role of Set7/9 in FOXO3-dependent ROS response

The FOXO (Forkhead O) family of transcriptional factors, comprised of FOXO1, FOXO3, FOXO4, and FOXO6, exerts anti-proliferative and pro-apoptotic functions in cells. These functions are executed via the transcriptional activation of several cell cycle and apoptotic regulators including p27, p21, p130, BIM, PUMA, and others 70, 71. Importantly, FOXO3 is also a critical regulator of NFkB activity. Through physical interaction with the latter, FOXO3 prevents NFkB from undergoing nuclear translocation, and thus attenuates NF-kB-mediated pro-inflammatory response 72. Furthermore, the FOXO family members participate in the regulation of autophagy, senescence, DNA-damage response, and drug resistance 73, 74. Another key function of FOXO transcription factors is their participation in the oxidative stress response. They do so by regulating the expression of antioxidant genes and by affecting the protein quality control. In this respect, FOXO3 is known to play one of the leading roles in the regulation of the anti-oxidative transcription program. Under genotoxic stress conditions, including high ROS concentrations, FOXO3 undergoes phosphorylation by several stress kinases such as JNK, MST1, and AMPK 75-77, resulting in its activation as a transcription factor. However, phosphorylation can have a dual effect on FOXO3: besides activation by kinases, as mentioned above, FOXO3 can also be phosphorylated by such kinases as AKT, ERK, and IKK that inhibit its activity. Mechanistically, they promote the export of FOXO3 from the nucleus, followed by its retention in the cytoplasm and subsequent ubiquitin-dependent degradation in proteasomes 78-80. Acetylation of FOXO3 by p300/CBP and deaсetylation by sirtuins (Sirt1, Sirt2 and Sirt3) also provide a fine-tuning regulatory mechanism of FOXO3 activity depending on the cellular context and types of stress stimuli 81-83. In turn, activated FOXO3 induces the expression of its transcriptional targets that control cell cycle arrest (p21, p27, p130) 84, 85, neutralization of ROS (CAT and MnSOD) 81, 86, 87, and induction of apoptosis (BIM, PUMA) 70. Among all the regulatory post-translational modifications, FOXO3 also undergoes lysine methylation mediated by Set7/9. Importantly, methyltransferase Set7/9 is involved in regulation of the FOXO3-mediated pathway at different stages.

### Set7/9 methylates the FOXO3 protein

It was shown that Set7/9 methylates FOXO3 at lysine 270 (K270) *in vitro* and *in vivo* and this modification leads to the inhibition of its DNA‐binding activity and subsequent transactivation of the target genes 88. This study demonstrated a correlation between the Set7/9-mediated methylation of FOXO3 and the reduction of ROS-induced apoptosis in neuronal cells. The authors have also shown that K270 methylated FOXO3 displayed a reduced trans-activation potential towards its target pro-apoptotic gene, *BCL2L11* (BIM) 88. However, the potential effects of the Set7/9-mediated methylation of FOXO3 on its target antioxidant genes, MnSOD and CAT, were not investigated in this study. Intriguingly, in another study, Calnan et al. reported that FOXO3 was methylated by Set7/9 on K271 55. This modification destabilized the protein in human cells but at the same time, increased its transactivation potential as judged from the luciferase assay results 55 (Figure 1B). This apparent contradiction may be resolved by considering the notion that posttranslational modifications of FOXO3 induced by ROS, namely acetylation/deacetylation by p300/CBP and sirtuins, have different effects on pro-apoptotic and antioxidant gene activation. Accordingly, it was shown that deacetylation of FOXO3 by Sirt1 and Sirt3 enhanced the FOXO3-mediated transactivation of genes responsible for cell cycle arrest, DNA repair, and ROS neutralization, along with the attenuation of pro-apoptotic genes 81, 89, 90. We speculate that Set7/9-mediated methylation of FOXO3 may also have a dual effect on the expression of its target genes, especially because p300/CBP and Set7/9 compete for the same target K271 lysine residue for acetylation and methylation, respectively.

Notably, Set7/9 was shown to interact with, and methylate, histone deacetylase Sirt1 at four positions (lysines 233, 235, 236, and 238) 54. Methylation by Set7/9 negatively regulates Sirt1 deacetylation activity. This finding, together with other observations 66, 91, suggests that Set7/9 might contribute to the FOXO3 regulation indirectly, through repression of the Sirt1 deacetylation activity (Figure 1B).

### Set7/9 stimulates the expression of E2F1 that in turn, inhibits FOXO

Another layer of the Set7/9-dependent regulation of FOXO3 is executed via interplay between Set7/9 and the negative regulator of FOXO3, transcription factor E2F1. Mechanistically, the DNA-binding region of E2F1 interacts with the transcription-activation domain of FOXO3 and thereby attenuates its transcriptional activity 92. Consequently, E2F1 attenuates the FOXO3-mediated expression of the MnSOD and CAT mRNAs. Importantly, Set7/9 was shown to be a critical co-activator of E2F1-dependent transcription 93. Expression levels of both E2F1 and Set7/9 define cell-cycle progression of tumor cells through the G1/S checkpoint upon DNA damage. Set7/9 affected the transcriptional activity of E2F1 through indirect modulation of histone modifications in the promoters of E2F1-dependent genes, thereby differentially affecting E2F1 transcription targets. Specifically, Set7/9 expression augmented the E2F1 activity towards the *CCNE1* (Cyclin E1) gene thereby promoting cell proliferation and on the contrary, repressed the E2F1-dependent expression of the *TP73* gene resulting in attenuated apoptosis 94. It is tempting to speculate that Set7/9-mediated methylation of E2F1 may also increase its binding to the FOXO3 promoter and down-regulation of the anti-oxidative damage response (Figure 1B).

## Set7/9-mediated regulation of Nrf2

**Figure 2:**
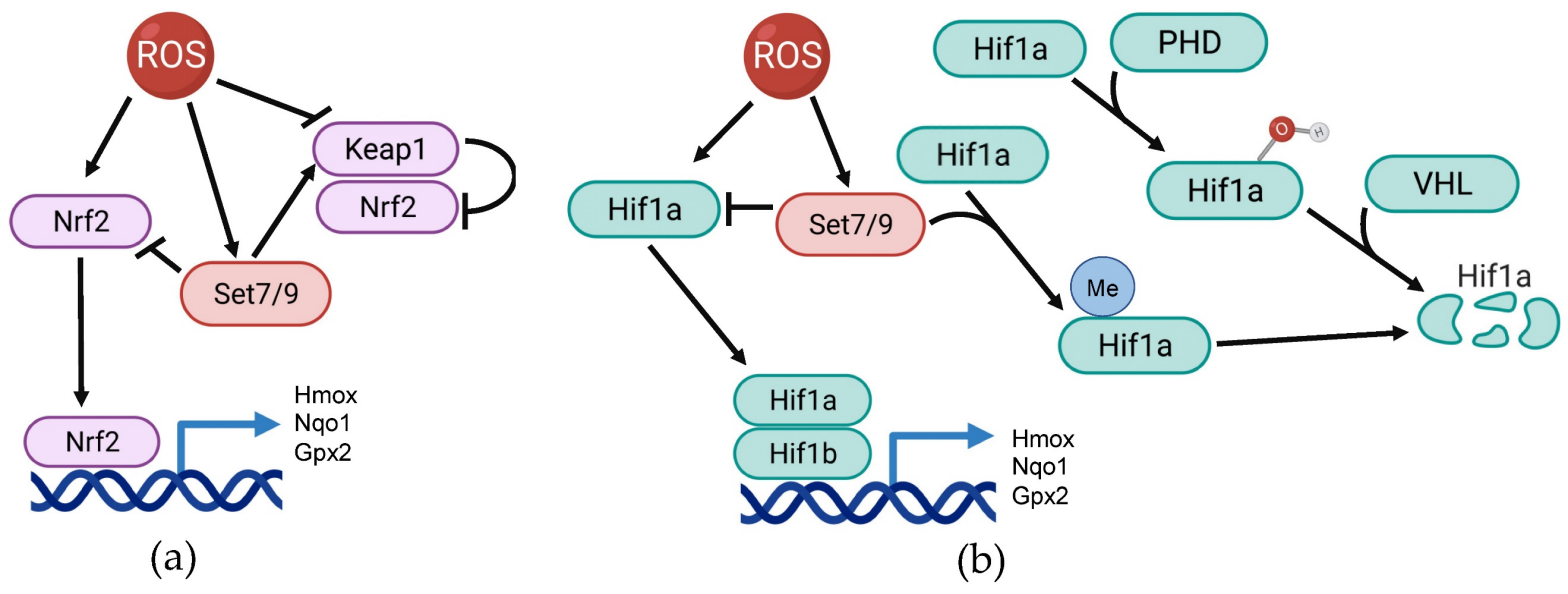
The scheme illustrating the participation of Set7/9 in regulation of Nrf2 and Hif1a in the context of ROS response. **(a)** Set7/9 inhibits Nrf2 activity as well as promotes activity of Keap1; **(b)** Set7/9 attenuates Hif1a expression and methylates Hif1a that promotes its proteasomal degradation.

The Nrf2 protein encoded by the corresponding *NFE2L2* gene is one of the key transcription factors sensing ROS accumulation and other stress stimuli. Being ubiquitously expressed in different cell types and tissues it is retained in inactive form in the cytoplasm by complexing with its negative regulator, Keap1. Oxidative stress or other stress stimuli induce the dissociation of the Nrf2-Keap1 complex, resulting in Nrf2 nuclear translocation and subsequent binding to the antioxidant response element (ARE) in the regulatory region of the target genes. The latter event results in transactivation of genes coding for antioxidant and cytoprotective enzymes such as heme oxygenase (Hmox1), Nqo1, glutathione peroxidase (GPX2) and many others (reviewed in 73, 74) (Figure 2A).

Set7/9 was shown to downregulate the expression of Nrf2 and its downstream targets under oxidative stress conditions in lung non-cancer epithelial cells Beas-2B 95. Furthermore, it was also demonstrated that Set7/9 physically interacted with Nrf2, but the physiological effect of this interaction remains unclear. Additionally, this study revealed the role of Set7/9 in the regulation of two additional ROS-sensitive factors - PGC-1α and NFkB. The PGC-1α protein plays the role of the co-activator for transcriptional factors participating in ROS neutralization such as FOXO and Nrf2, and also upregulates Sirt3 (reviewed in 96). Thus, Set7/9 inhibits the key regulator of mitochondrial function and biogenesis, PGC-1α. Furthermore, according to this study, Set7/9 was shown to act, in contrast to PGC-1α, as a positive regulator of NFkB, hence activating the NFkB-dependent pro-inflammatory gene expression program. On the contrary, suppression of Set7/9 augmented the expression of antioxidant genes and subsequent ROS neutralization in response to H_2_O_2_ treatment 95. In a more recent study, Dang et al. using cardiomyocytes exposed to hypoxia/reoxygenation, demonstrated that the silencing of Set7/9 resulted in Nrf2 stabilization and subsequent chromatin acetylation within the regulatory regions of genes coding for Heme Oxygenase 1 (*HMOX1*) and NAD(P)H:quinone acceptor oxidoreductase (*NQO1*). This was concomitant with the reduction of ROS accumulation and caspase-3 activity 97. In contrast, the study performed on human prostate cancer (PC) cells demonstrated that the knockdown of *SET7/9* attenuated the expression of Nrf2 antioxidant targets - Nqo1 and glutathione S-transferase theta 2 (Gstt2) expression in response to H_2_O_2_ treatment. Consistently, in Set7/9-deficient PC cells an increased production of ROS was observed. Moreover, H_2_O_2_ treatment caused much more severe DNA damage in Set7/9 KD cells compared to normal cells. Although the attenuation of Set7/9 did not affect Nrf2 expression, the authors hypothesized that the observed effects were mediated via Nrf2 whose expression was regulated by Set7/9-mediated methylation of histones in the promoter region of *NFE2L2* 98.

Thus, it can be assumed that the mechanism of Set7/9-mediated regulation of Nrf2 can be tissue-specific and therefore may require unique accessory factors.

## Set7/9 and ROS/Hif1a pathway

It has been firmly established that hypoxia and oxidative stress are tightly connected. In fact, hypoxiс conditions were shown to promote ROS accumulation 99. The transcription factor Hif1 is a master regulator of cellular transcriptional response to hypoxic conditions. Hif1 exerts its functions by binding to the hypoxia response elements (HRE) situated within the regulatory elements of its target genes. It promotes oxygenation and metabolic adaptation to hypoxia through the activation of numerous downstream targets, thereby affecting glucose metabolism, angiogenesis, cellular growth and survival, invasion and metastasis 100. Hif1 acts as heterodimer comprised of two subunits - oxygen-regulated subunits Hif1a/Hif2a and a constitutively expressed subunit, Hif1b. Notably, the activity of the Hif1 heterodimer is mostly controlled at the level of stability and expression of Hif1a and Hif2a subunits 101.

In the absence of activating stimuli, the stability of Hif1a is regulated by hydroxylase domain proteins (PHDs) and the von Hippel-Lindau tumor suppressor protein (VHL). The PHD-containing proteins covalently modify Hif1a via hydroxylation of proline residues 402 and 564. This modification stimulates the binding of Hif1a and the VHL ubiquitin ligase, which results in the subsequent degradation of Hif1a in proteasomes 102. Interestingly, the expression of the Hif1a-coding gene is regulated by ROS 23. At the molecular level, Nrf2 binds to the ARE element within the *HIF1A* gene in response to the rising level of ROS.

Upon activation of oxidative stress, Hif1a enhances the expression of several key glycolytic regulators such as Glut1, LDHA, and aldolases. Shifting the cellular energy metabolism from oxidative phosphorylation (OXPHOS) to aerobic glycolysis (the Warburg effect) attenuates the level of ROS-induced stress 3, 103. Notably, Hif1a stabilization can also be mediated by changes in proteolytic activity of the proteasome, which is regulated by post-translational modifications in response to ROS 69, 104.

Importantly, two research groups demonstrated that Set7/9-mediated methylation of Hif1a under hypoxic conditions increased the proteasomal degradation of Hif1a and hence prevented transactivation of Hif1 target genes 105, 106 (Figure 2B).

In line with this, we have recently showed that the genetic suppression of Set7/9 has led to the upregulation and activation of Hif1a and its target glycolytic genes even under normoxic conditions. Set7/9 inhibition increased the level of glycolysis and subsequent reduction of ROS accumulation in human lung cancer cells 107. Thus, collectively, it can be concluded that Set7/9 antagonizes Hif1 activity both under normoxic and hypoxic conditions.

## The potential role of Set7/9 in c-Myc-mediated regulation of ROS levels

**Figure 3:**
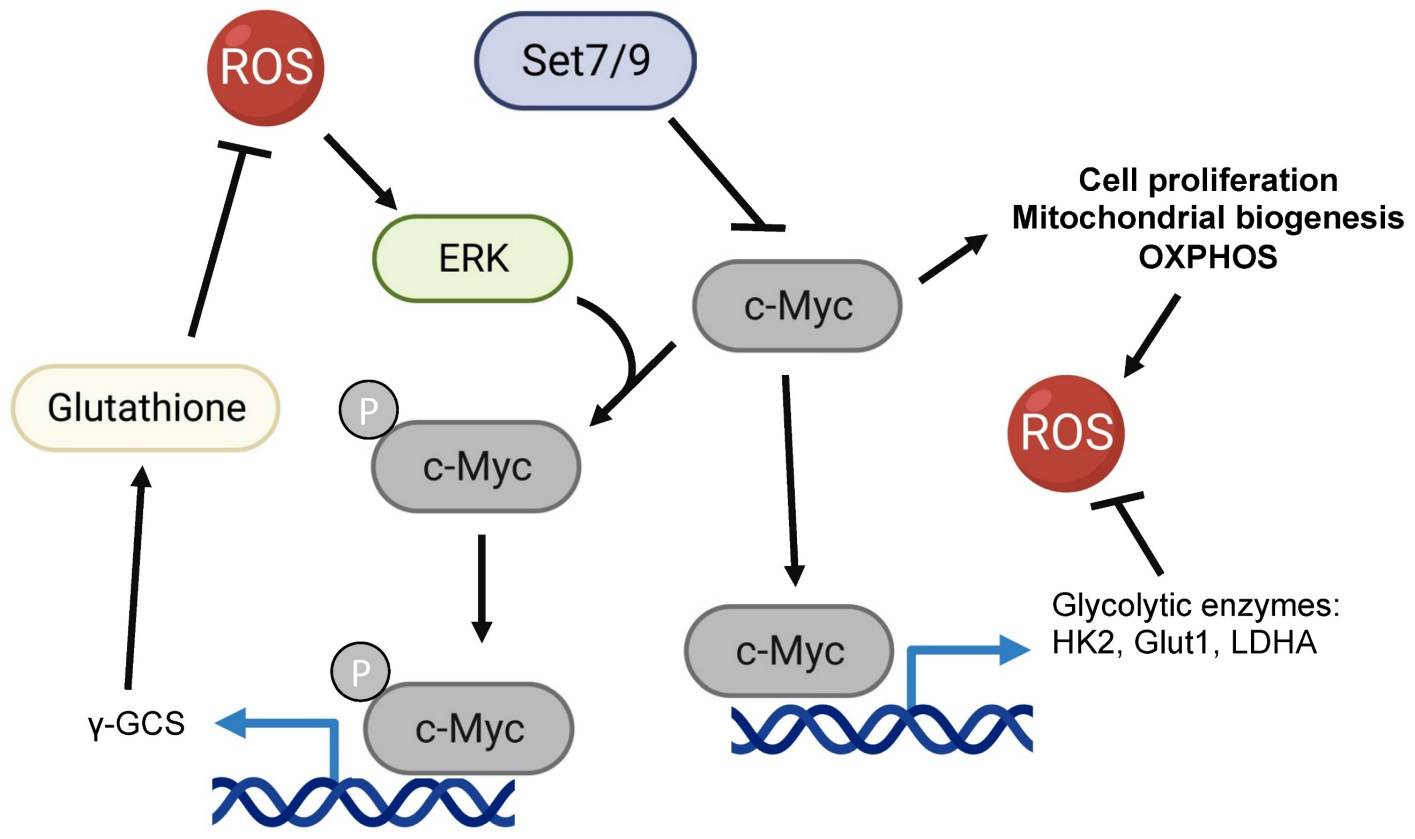
The potential role of Set7/9 in coordination of c-Myc-dependent ROS accumulation and oxidative stress response.

The oncogenic protein, c-Myc, is a known transcriptional regulator whose numerous gene targets participate in a coordinated fashion in many cellular processes, including metabolism and proliferation. Accordingly, c-Myc also plays a role in the ROS production and oxidative stress response.

c-Myc was repeatedly shown to promote ROS production via the activation of cellular proliferation, mitochondrial biogenesis, and oxidative phosphorylation 108-110 (Figure 3). At the same time, c-Myc is one of the two currently known (along with Hif1a) transcriptional regulators of glycolytic enzymes HK2, Glut1 (SLC2A), LDHA, and others 111-115. As it was mentioned in the previous section, the shift of the balance from OXPHOS towards glycolysis leads to a reduction of ROS accumulation 3, 103 (Figure 3).

Interestingly, in response to oxidative damage c-Myc was shown to be phosphorylated by ERK kinase. The latter increases the c-Myc-mediated expression of γ-GCS (γ-glutamyl-cysteine synthetase) and glutathione (GSH) biosynthesis 116. Being one of the key antioxidants, GSH acts as an intracellular hydrogen peroxide and free-radical-neutralizing enzyme 116. In this respect, our recent work suggests that Set7/9 is a negative regulator of c-Myc-dependent glycolytic targets and hence may contribute to c-Myc-mediated ROS response 107 (Figure 3).

## Set7/9 regulates PARP1 activity under ROS-inducing stress conditions

The product of the *ARTD1* gene, poly (ADP-ribose) polymerase PARP1, is a crucial enzyme carrying out PARylation (ADP-ribosylation) of various proteins including histones and non-histone targets. Besides the oxidative stress response, PARP1 plays crucial roles in key cellular and physiological processes such as chromatin remodeling, inflammation, DNA repair, regulation of transcription, as well as induction of cell death (reviewed in 117).

**Figure 4:**
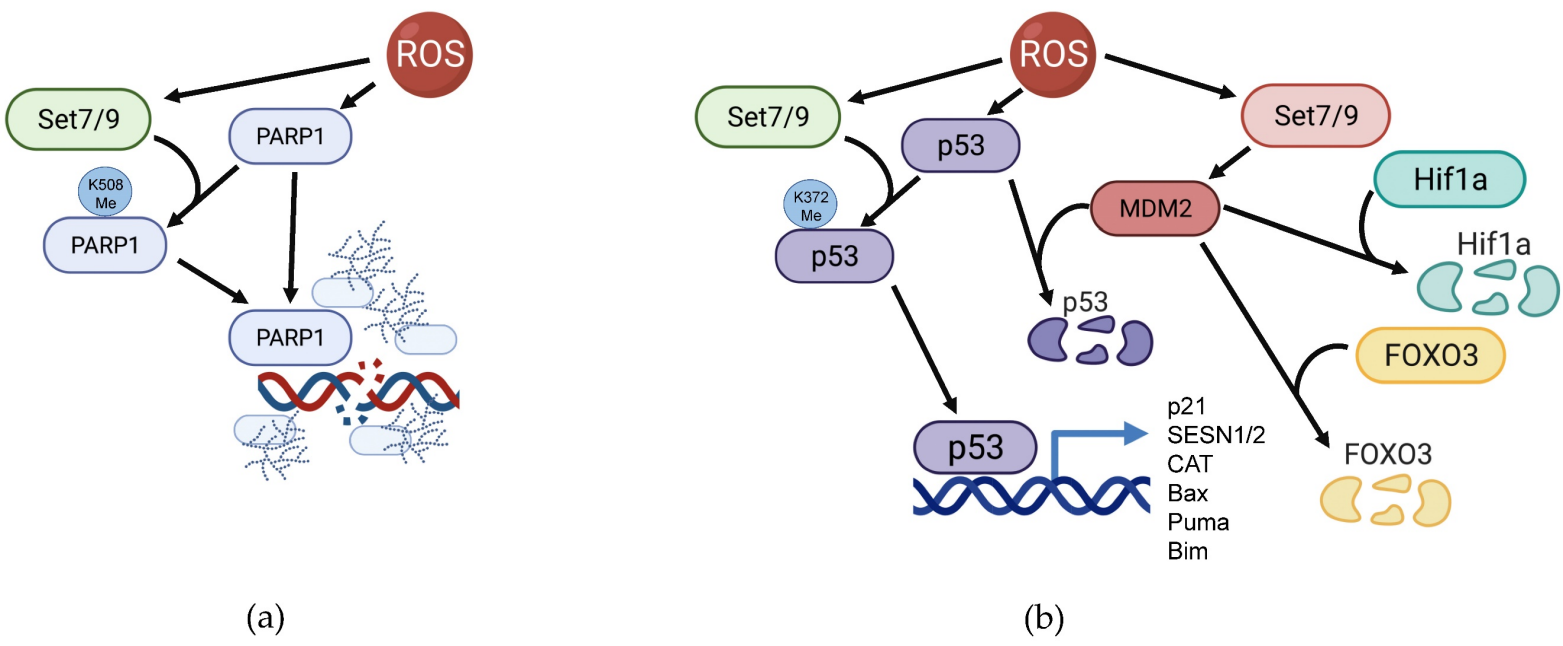
Participation of Set7/9 in regulation of PARP1 and p53 in the context of ROS response. **(a)** Set7/9 methylates PARP1 on K508 thereby facilitating its accumulation at the sites of DNA breaks and promoting its enzymatic activity towards its protein targets; **(b)** Set7/9 activates p53 by methylating it on K372 and thereby promoting MDM2 expression.

It was repeatedly shown that ROS accumulation activates PARP1 as well as augments the overall PARylation level of cellular proteins 118-121. PARP1 plays the role of a cellular rheostat by gauging the level of ROS-inflicted DNA damage in the cell: if the number of clustered DNA lesions generated by ROS is rather moderate, then a small increase of PARP1 expression promotes base excision repair (BER) of the damaged DNA. However, the excessive oxidative stress leads to overactivation of PARP1 thereby promoting different types of cell death including apoptosis 122, 123 and necrosis 124. The latest issue of Cell Death Nomenclature distinguishes a special cell death type that depends on the PARP1 activation - parthanatos 125. Conversely, the suppression of PARP1 activity mostly leads to enhanced survival of cells under oxidative stress 120, 123, 126, 127. Notably, Set7/9 was shown to interact with and methylate PARP1 at K508. This modification positively regulates PARP1 recruitment to sites of DNA damage and augments its enzymatic activity in response to oxidative stress 45 (Figure 4A). Thus, it is tempting to speculate that Set7/9 is able to induce parthanatos at excessive ROS amounts.

## Other scenarios of Set7/9-mediated regulation of ROS-induced signaling

One of the critical developmental pathways, the WNT/β-catenin signaling cascade was shown to be indirectly activated by ROS via the regulation of β-catenin by the ROS-sensing protein peridoxin 128, 129. The opposite is also true: WNT pathway regulates ROS production during embyogenesis by one of the targets of Wnt5 activation, Nox (reviewed in 130). Importantly, upon aberrant activation this pro-proliferative and pro-survival pathway also plays a crucial role in tumor transformation 131. In this respect, it has been demonstrated that Set7/9 binds to and methylates β-catenin, thereby facilitating its degradation via the (GSK)-3-mediated phosphorylation 132. Conversely, Set7/9 ablation was shown to promote cell proliferation via the upregulation of WNT/β-catenin target genes, *CCND* and *MYC*. Importantly, Set7/9-dependent methylation of β-catenin is enhanced under oxidative stress conditions, thus forming a regulatory mechanism for the WNT/β-catenin signaling during the oxidative stress response 132. Taken together, Set7/9 appears to be an important regulator of β-catenin under stress.

Another important connection between Set7/9 and ROS is mediated by the tumor suppressor protein, p53. The p53 protein is a transcription factor that is stabilized under stress conditions by numerous post-translational modifications 133, including methylation by Set7/9 49, 134, 135. Once stabilized, p53 transactivates a plethora of target genes whose products are involved in various cellular processes, including cell cycle arrest and apoptosis 136-140. According to the current paradigm, p53 is responsible for the activation of cell-cycle arrest and DNA repair through transcriptional activation of the respective genes. In the case of inefficient DNA repair, p53 triggers apoptosis via transcriptional induction of pro-apoptotic genes. In response to ROS, p53 additionally stimulates the expression of several genes coding for antioxidant enzymes such as SESN1/2, MnSOD, GPX, and others 141-143. In line with this, the antioxidant, cytoprotective and pro-survival roles of p53 was demonstrated by Ding et al. 144. However, it was shown that if the amount of ROS-induced DNA damage is insurmountable, p53 activates PARP1-dependent cell death 145.

Set7/9 positively regulates p53 activity on several levels. First, Set7/9 methylates p53 at K372 and this modification results in p53 stabilization, nuclear accumulation and enhanced transactivation of target genes 40 (Figure 4B). Set7/9-mediated K372 methylation of p53 prevents the inhibitory methylation at the neighboring lysine residue K370 carried out by another KMT, SMYD2 146. Furthermore, K372 methylation enables TIP60-dependent acetylation of p53 at K120 required for transactivation of p53-dependent pro-apoptotic genes 147. Sirt1 deacetylates and represses p53 under genotoxic stress 148. As it was mentioned above, Set7/9 also mediates inhibitory methylation of Sirt1 deacetylase that downregulates its enzymatic activity. Consequently, Set7/9-dependent methylation of Sirt1 prevents the Sirt1-p53 interaction and hence promotes p53 activity 106.

MDM2 is an E3 ubiquitin ligase, whose corresponding gene is under the control of p53. In turn, Mdm2 keeps p53 at low levels, thereby forming a negative feed-back loop 49. In fact, preventing the interaction between p53 and Mdm2 by small molecule inhibitors results in a robust nuclear accumulation of p53 and the onset of apoptosis 140, 149, 150. We have shown that Set7/9 attenuates MDM2 expression and suppresses MDM2 functions under DNA damage conditions 151 (Figure 4B). In addition to p53, MDM2 is shown to promote the ROS-induced degradation of Hif1a 152 and ubiquitination of FOXO3 79, 153 (Figure 4B). Moreover, MDM2 promotes p65/RelA expression and acts as its transcriptional co-activator through various mechanisms 154. Taken together, it is tempting to speculate that by affecting MDM2, Set7/9 also attenuates the expression of ROS-inducible RelA/MDM2 target genes.

## The *in vivo* role of Set7/9 in ROS-associated pathological processes

Considering the number of connections between Set7/9 and ROS regulatory proteins, it is not surprising that this methyltransferase was found to be an important contributor to ROS-associated pathological processes also at the organismal level.

Despite the fact that *SETD7* knockout mice demonstrated no developmental abnormalities, disruption of Set7/9 causes developmental disorders in the heart and skeletal muscles in zebrafish embryos and malformation of pancreas during *Xenopus* embryogenesis 155-158. Given the complexity of mutually compensatory regulatory cues in response to ROS signaling in mammals, we suggest that the effect of Set7/9 malfunctioning at the level of the whole organism may differ from that observed *in cellulo.* Therefore, a lack of gross phenotypic changes that can be directly attributed to the mis-regulation of Set7/9 in the model animals indicates that the latter likely plays a fine-tuning role in the ROS response and therefore makes Set7/9 a promising target for pharmacological intervention to mitigate ROS-related pathological conditions.

Diabetes mellitus (DM) is one of the most common diseases associated with altered ROS production and metabolism. Indeed, hyperglycemia is known to lead to ROS accumulation and oxidative stress in different tissues and organs 159. Type 1 diabetes (T1D) is an autoimmune disease in which pancreatic beta cells are destroyed by autoreactive T cells. The transcription factor NF-κB has been widely studied for its role in development of type 1 diabetes. Recent data have shown that NF-κB is required for the activation of autoreactive T cells, and its hyperactivity in monocytes and dendritic cells alters cytokine secretion and antigen presentation, thereby ultimately contributing to the initiation of T1D. Type 2 diabetes (T2D) is mediated by the inability of muscle, liver, and fat cells to adequately respond to insulin signaling, which normally results in blocking the release of glucose. Persisting high glucose levels can induce nephropathy, retinopathy, cardiomyopathy, and neuropathy. In T2D cells, activated NF-κB was demonstrated to induce both apoptosis of beta-cells and insulin resistance. Furthermore, ROS and advanced glycation end products (AGEs) exacerbate the complications of DM. Age-related DM often associates with methylation of DNA and epigenetic changes, including histone methylation 160, 161.

In this respect, Set7/9 was repeatedly shown to be overexpressed in islets of rodent and human pancreas 162-165. Using the murine model with β cells-specific knock-out of Set7/9 it was demonstrated that the ablation of Set7/9 decreased mRNA levels of *PDX1, GLUT2, INS1,* and* INS2* genes required for glucose-stimulated insulin secretion 166. Indeed, mice with attenuated Set7/9 expression in β cells demonstrated impaired insulin secretion and glucose metabolism 163, 166. Additionally, a recent study demonstrated that Set7/9 is a transcriptional target of peroxisome proliferator-activated receptor PPARγ in β cells of mice. The enhancement of PPARγ-mediated Set7/9 expression was observed upon glycemic adaptation in partial pancreatectomy rat models 165. These data indicate that Set7/9 is required for an appropriate glucose sensing and insulin production by islet β cells.

The study by Mirmira's group argued that Set7/9 was directly responsible for maintaining increased levels of H3K4 di-methylation in the promoter regions of *INS1/2* and *GLUT2* genes in pancreatic β cells. The increased level of H3K4 methylation was concomitant with high levels of transcription of the corresponding genes 163, 167. Again, as KMTase that mono-methylates its target lysines it is unlikely that Set7/9 can directly mediate di-methylation of H3K4. The exact molecular mechanism behind this phenomenon requires additional elucidation. However, altered functions of islet cells and changes in glucose metabolism are indeed tightly correlated with ROS-related diseases, which may tie together Set7/9, glucose metabolism, and ROS.

In another study Set7/9 has been shown to upregulate the expression of a monocyte chemoattractant protein MCP-1 (encoded by the *CCL2* gene) in the kidneys of diabetic mice 168. Being a component of the NFkB inflammatory cascade, MCP-1 is known to be one of the key factors that initiate hyperglycemia-induced inflammation in the kidneys, leading to fibrosis and subsequent renal failure. Set7/9 was shown to upregulate *CCL2* through H3K3 methylation of the promoter region. Interestingly, the authors of that study uncovered that the ER stress-inducible transcription factor XBP1 was responsible for the increased Set7/9 expression in the kidneys of diabetic mice 168.

This study is consistent with the data obtained by Sharma et al. demonstrating the elevation of Set7/9 expression in ischemic renal injury (IRI) rat models 169. Indeed, it was shown that IRI increased the level of Set7/9, H3K4 methylation, and MCP-1 expression, as well as activating the NFkB inflammatory cascade in ischemic kidney tissues of both diabetic and non-diabetic rats 169. Importantly, the administration of the Set7/9 inhibitor cyproheptadine significantly improved the renal functioning of IRI rats.

The involvement of Set7/9 in the development of diabetic nephropathy was also convincingly demonstrated in the study by Sasaki et al. 170. There, Set7/9 was identified as a transcription target of TGF- β1 in renal cells and kidney tissues of mice with induced obstructive nephropathy 170. Importantly, an injection of Set7/9 siRNA attenuated TGF-β1-induced fibrogenesis in this animal disease model. Additionally, using the histological material from the patients who underwent a renal biopsy, it was revealed that Set7/9 expression was associated with the severity of fibrosis in the human kidney. Finally, this study demonstrated that the small molecule inhibitor of Set7/9, sinefungin, ameliorated renal fibrosis in the unilateral ureteral obstruction (UUO) of mice and inhibited the TGF-β1-induced α-SMA expression in renal cells 170.

Another important role of Set7/9 in ROS-dependent processes was demonstrated in myocardial ischemic injury. Myocardial ischemia/reperfusion injury (MIRI) is a frequently observed medical condition associated with several cardiovascular diseases including coronary heart disease. MIRI is strongly associated with ROS-derived damage of tissues both at the ischemic and reperfusion stages observed in 171, 172. Thus, rapid ROS accumulation is being observed under hypoxic conditions resulting from mitochondrial disfunction and a decrease of intracellular pH. However, increased oxygenation as a result of reperfusion also causes the generation of ROS, thereby reinforcing oxidative stress.

**Figure 5:**
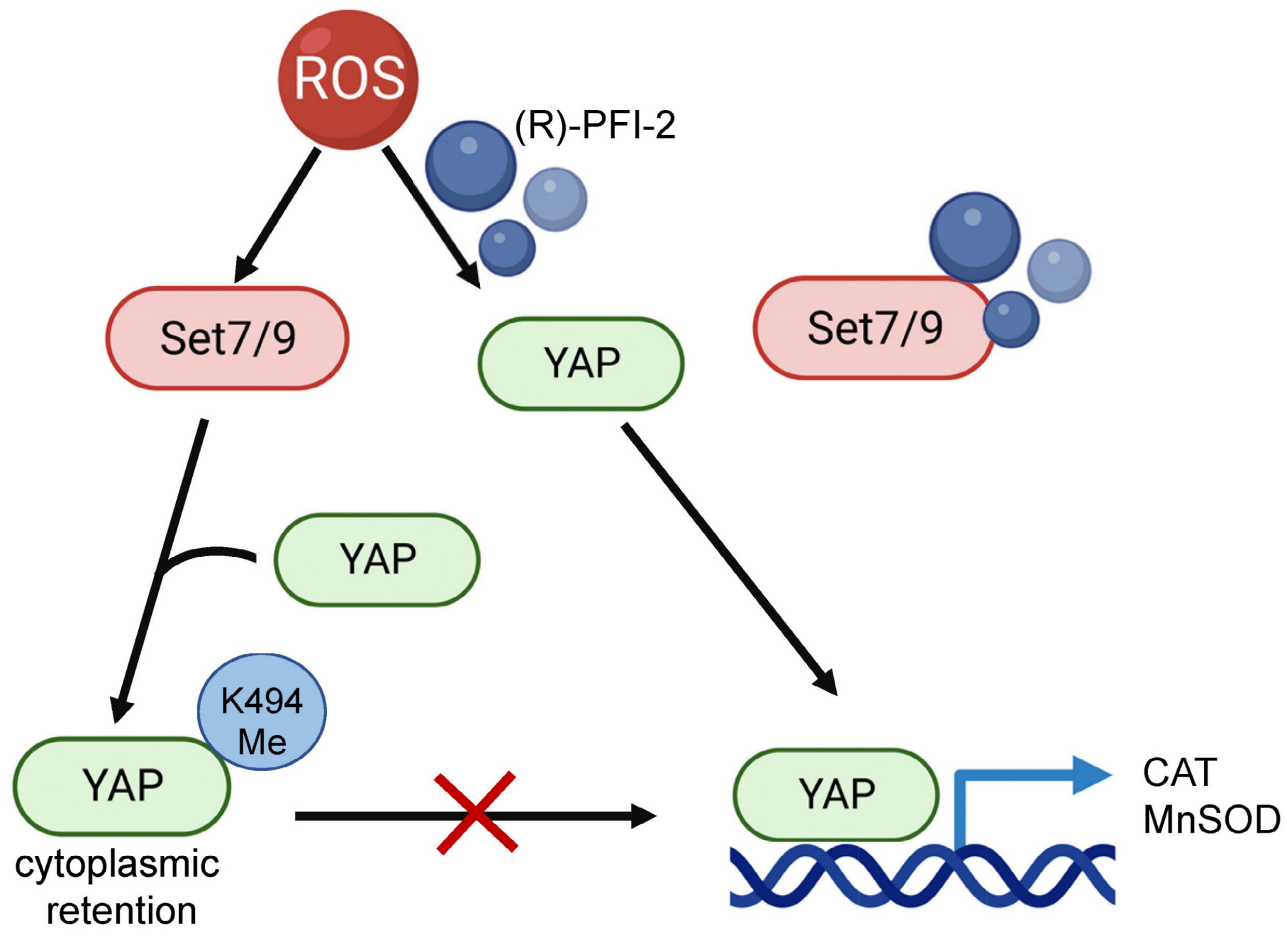
The effect of Set7/9-driven methylation of YAP on transcription of its target genes, CAT and MnSOD. Set7/9 methylates YAP at K494 that results in retention of YAP in the cytoplasm. Inhibiting Set7/9 using a small-molecule inhibitor (R)-PFI-2 prevents YAP methylation, restores its nuclear localization and stimulates expression of YAP target genes, CAT and MnSOD.

Using Set7/9 knockout mice it was shown that the depletion of Set7/9 attenuated MIRI, ROS accumulation, and left ventricular dysfunction. On the molecular level, this was achieved by restoring the YAP-dependent transcription of MnSOD and CAT in Set7/9 knockout cells 173. Being a methyltransferase for YAP transcription factor Set7/9 causes YAP cytoplasmic retention and attenuates the expression of its transcription targets 43. In line with this, inhibition of Set7/9 using small molecule inhibitor (R)-PFI-2 was shown to restore nuclear localization of YAP and transcriptional activation of YAP-dependent genes coding for antioxidant enzymes MnSOD and CAT in neonatal rat ventricular myocytes 173 (Figure 5). Consistent with this hypothesis, elevated levels of Set7/9 expression were detected in peripheral blood mononuclear cells of patients with ST-elevation myocardial infarction (STEMI) 173. Interestingly, this work resonates with the aforementioned *in vitro* study demonstrating the protective effect of Set7/9 inhibition in cardiomyocytes subjected to hypoxia-reoxygenation achieved through Nrf2 stabilization and activation of antioxidant genes 97.

Collectively, these *in vivo* studies demonstrate the important role of Set7/9 in the development of ROS-associated pathological conditions. This experimental evidence points to the pharmacological inhibition of Set7/9 as a valid strategy for treatment of these conditions.

## Conclusions

ROS-induced signaling cascades are critical in determining the fate of a cell. ROS can cause irreversible changes in cellular physiology in the event of its excessive accumulation, and/or malfunctioning of the ROS neutralizing system. Here we presented the published data concerning the important role of Set7/9 in ROS response. The fact that Set7/9 regulates this process at different levels and with different outcomes, raises the necessity to consider the status of Set7/9 when designing novel therapeutic approaches to correct the effect of ROS imbalance. In line with this notion is the fact that methyltransferases-specific inhibitors are now being considered as a promising approach for the treatment of such ROS-dependent diseases as cancer, renal, and cardiovascular disorders 174-176. Importantly, a number of small molecule inhibitors for other methyltransferases, e. g. EZH2, PRMT1/5, DOT1L, are being examined as anticancer drugs at different stages of clinical trials 177, 178. In this respect, it should be noted that there are several Set7/9-specific inhibitors developed recently that may hold promise as new drugs for treating ROS-inflicted diseases. One of them, (R)-PFI-2, inhibits the methyltransferase activity of Set7/9 at nanomolar concentrations and does not exhibit toxicity at an effective concentration in human cells 179, 180. The two others, 119913-X and 610930-N, were identified via high-throughput screening and require additional characterization 181. Thus, pharmacological modulation of Set7/9 activity may prove an effective approach to treat oxidative stress-related diseases.

## References

[1] D'Autréaux B, Toledano MB (2007). ROS as signalling molecules: mechanisms that generate specificity in ROS homeostasis. Nature reviews Molecular cell biology.

[2] Nugud A, Sandeep D, El-Serafi AT (2018). Two faces of the coin: Minireview for dissecting the role of reactive oxygen species in stem cell potency and lineage commitment. Journal of advanced research.

[3] Ryu JM, Lee HJ, Jung YH, Lee KH, Kim DI, Kim JY (2015). Regulation of stem cell fate by ROS-mediated alteration of metabolism. International journal of stem cells.

[4] Davalli P, Mitic T, Caporali A, Lauriola A, D'Arca D (2016). ROS, cell senescence, and novel molecular mechanisms in aging and age-related diseases. Oxidative medicine and cellular longevity. 2016.

[5] Shekhova E (2020). Mitochondrial reactive oxygen species as major effectors of antimicrobial immunity. PLoS pathogens.

[6] Li Z, Wu J, DeLeo CJ (2006). RNA damage and surveillance under oxidative stress. IUBMB life.

[7] Shah D, Sah S, Nath SK (2013). Interaction between glutathione and apoptosis in systemic lupus erythematosus. Autoimmunity reviews.

[8] Zhou D, Shao L, Spitz DR (2014). Reactive oxygen species in normal and tumor stem cells. Advances in cancer research: Elsevier.

[9] Yu T, Sheu S-S, Robotham JL, Yoon Y (2008). Mitochondrial fission mediates high glucose-induced cell death through elevated production of reactive oxygen species. Cardiovascular research.

[10] De Jager T, Cockrell A, Du Plessis S (2017). Ultraviolet light induced generation of reactive oxygen species. Ultraviolet Light in Human Health, Diseases and Environment.

[11] Storz P (2006). Reactive oxygen species-mediated mitochondria-to-nucleus signaling: a key to aging and radical-caused diseases. Science's STKE.

[12] Kumsta C, Jakob U (2009). Redox-regulated chaperones. Biochemistry.

[13] Srinivas US, Tan BW, Vellayappan BA, Jeyasekharan AD (2019). ROS and the DNA damage response in cancer. Redox biology.

[14] Redza-Dutordoir M, Averill-Bates DA (2016). Activation of apoptosis signalling pathways by reactive oxygen species. Biochimica et Biophysica Acta (BBA)-Molecular Cell Research.

[15] Kranner I, Birtić S, Anderson KM, Pritchard HW (2006). Glutathione half-cell reduction potential: a universal stress marker and modulator of programmed cell death?. Free Radical Biology and Medicine.

[16] Ortega AL, Mena S, Estrela JM (2011). Glutathione in cancer cell death. Cancers.

[17] Alfadda AA, Sallam RM (2012). Reactive oxygen species in health and disease. Journal of Biomedicine and Biotechnology. 2012.

[18] El-Kenawi A, Ruffell B (2017). Inflammation, ROS, and mutagenesis. Cancer cell.

[19] Hoffmann MH, Griffiths HR (2018). The dual role of Reactive Oxygen Species in autoimmune and inflammatory diseases: evidence from preclinical models. Free Radical Biology and Medicine.

[20] Newsholme P, Cruzat VF, Keane KN, Carlessi R, de Bittencourt Jr PIH (2016). Molecular mechanisms of ROS production and oxidative stress in diabetes. Biochemical Journal.

[21] Yang S, Lian G (2020). ROS and diseases: Role in metabolism and energy supply. Molecular and cellular biochemistry.

[22] Lennicke C, Cochemé HM (2021). Redox metabolism: ROS as specific molecular regulators of cell signaling and function. Molecular Cell.

[23] Lacher SE, Levings DC, Freeman S, Slattery M (2018). Identification of a functional antioxidant response element at the HIF1A locus. Redox biology.

[24] Liu B, Chen Y, Clair DKS (2008). ROS and p53: a versatile partnership. Free Radical Biology and Medicine.

[25] Klotz L-O, Sánchez-Ramos C, Prieto-Arroyo I, Urbánek P, Steinbrenner H, Monsalve M (2015). Redox regulation of FoxO transcription factors. Redox biology.

[26] Bubici C, Papa S, Pham C, Zazzeroni F, Franzoso G (2006). The NF-kB-mediated control of ROS and JNK signaling. Histology and histopathology.

[27] Choi H-J, Kim S-J, Mukhopadhyay P, Cho S, Woo J-R, Storz G (2001). Structural basis of the redox switch in the OxyR transcription factor. Cell.

[28] Ray PD, Huang B-W, Tsuji Y (2012). Reactive oxygen species (ROS) homeostasis and redox regulation in cellular signaling. Cellular signalling.

[29] Zhang J, Wang X, Vikash V, Ye Q, Wu D, Liu Y (2016). ROS and ROS-mediated cellular signaling. Oxidative medicine and cellular longevity. 2016.

[30] Shadel GS, Horvath TL (2015). Mitochondrial ROS signaling in organismal homeostasis. Cell.

[31] Verbon EH, Post JA, Boonstra J (2012). The influence of reactive oxygen species on cell cycle progression in mammalian cells. Gene.

[32] Reczek CR, Chandel NS (2015). ROS-dependent signal transduction. Current opinion in cell biology.

[33] Kietzmann T, Petry A, Shvetsova A, Gerhold JM, Görlach A (2017). The epigenetic landscape related to reactive oxygen species formation in the cardiovascular system. British Journal of Pharmacology.

[34] Wu Q, Ni X (2015). ROS-mediated DNA methylation pattern alterations in carcinogenesis. Current drug targets.

[35] Yi X, Zhu Q-X, Wu X-L, Tan T-T, Jiang X-J (2022). Histone Methylation and Oxidative Stress in Cardiovascular Diseases. Oxidative Medicine and Cellular Longevity. 2022.

[36] Afanas' ev I (2014). New nucleophilic mechanisms of ros-dependent epigenetic modifications: comparison of aging and cancer. Aging & Disease.

[37] Morgunkova A, Barlev NA (2006). Lysine methylation goes global. Cell cycle.

[38] Park I-G, Jeon M, Kim H, Lee JM (2021). Coordinated methyl readers: Functional communications in cancer. Seminars in Cancer Biology: Elsevier.

[39] Ivanov GS, Ivanova T, Kurash J, Ivanov A, Chuikov S, Gizatullin F (2007). Methylation-acetylation interplay activates p53 in response to DNA damage. Molecular and cellular biology.

[40] Chuikov S, Kurash JK, Wilson JR, Xiao B, Justin N, Ivanov GS (2004). Regulation of p53 activity through lysine methylation. Nature.

[41] Subramanian K, Jia D, Kapoor-Vazirani P, Powell DR, Collins RE, Sharma D (2008). Regulation of estrogen receptor α by the SET7 lysine methyltransferase. Molecular cell.

[42] Gaughan L, Stockley J, Wang N, McCracken SR, Treumann A, Armstrong K (2011). Regulation of the androgen receptor by SET9-mediated methylation. Nucleic acids research.

[43] Oudhoff MJ, Freeman SA, Couzens AL, Antignano F, Kuznetsova E, Min PH (2013). Control of the hippo pathway by Set7-dependent methylation of Yap. Developmental cell.

[44] Ea C-K, Baltimore D (2009). Regulation of NF-κB activity through lysine monomethylation of p65. Proceedings of the National Academy of Sciences.

[45] Kassner I, Andersson A, Fey M, Tomas M, Ferrando-May E, Hottiger MO (2013). SET7/9-dependent methylation of ARTD1 at K508 stimulates poly-ADP-ribose formation after oxidative stress. Open biology.

[46] Kouskouti A, Scheer E, Staub A, Tora L, Talianidis I (2004). Gene-specific modulation of TAF10 function by SET9-mediated methylation. Molecular cell.

[47] Estève P-O, Chin HG, Benner J, Feehery GR, Samaranayake M, Horwitz GA (2009). Regulation of DNMT1 stability through SET7-mediated lysine methylation in mammalian cells. Proceedings of the National Academy of Sciences.

[48] Vasileva E, Shuvalov O, Petukhov A, Fedorova O, Daks A, Nader R (2020). KMT Set7/9 is a new regulator of Sam68 STAR-protein. Biochemical and Biophysical Research Communications.

[49] Marouco D, Garabadgiu AV, Melino G, Barlev NA (2013). Lysine-specific modifications of p53: a matter of life and death?. Oncotarget.

[50] Daks A, Vasileva E, Fedorova O, Shuvalov O, Barlev NA (2022). The Role of Lysine Methyltransferase SET7/9 in Proliferation and Cell Stress Response. Life.

[51] Gu Y, Zhang X, Yu W, Dong W (2022). Oncogene or Tumor Suppressor: The Coordinative Role of Lysine Methyltransferase SET7/9 in Cancer Development and the Related Mechanisms. Journal of Cancer.

[52] Morgan MJ, Liu Z-g (2011). Crosstalk of reactive oxygen species and NF-κB signaling. Cell research.

[53] Imbert V, Rupec RA, Livolsi A, Pahl HL, Traenckner EB-M, Mueller-Dieckmann C (1996). Tyrosine phosphorylation of IκB-α activates NF-κB without proteolytic degradation of IκB-α. Cell.

[54] Liu A, Zhang B, Zhao W, Tu Y, Wang Q, Li J (2021). Catalpol ameliorates psoriasis-like phenotypes via SIRT1 mediated suppression of NF-κB and MAPKs signaling pathways. Bioengineered.

[55] Rojo AI, Salinas M, Martín D, Perona R, Cuadrado A (2004). Regulation of Cu/Zn-superoxide dismutase expression via the phosphatidylinositol 3 kinase/Akt pathway and nuclear factor-κB. Journal of Neuroscience.

[56] Djavaheri-Mergny M, Javelaud D, Wietzerbin J, Besançon F (2004). NF-κB activation prevents apoptotic oxidative stress via an increase of both thioredoxin and MnSOD levels in TNFα-treated Ewing sarcoma cells. FEBS letters.

[57] Kairisalo M, Korhonen L, Blomgren K, Lindholm D (2007). X-linked inhibitor of apoptosis protein increases mitochondrial antioxidants through NF-κB activation. Biochemical and biophysical research communications.

[58] Ali F, Sultana S (2012). Repeated short-term stress synergizes the ROS signalling through up regulation of NFkB and iNOS expression induced due to combined exposure of trichloroethylene and UVB rays. Molecular and cellular biochemistry.

[59] Huang C-Y, Lee C-H, Tu C-C, Wu C-H, Huang M-T, Wei P-L (2018). Glucose-regulated protein 94 mediates progression and metastasis of esophageal squamous cell carcinoma via mitochondrial function and the NF-kB/COX-2/VEGF axis. Oncotarget.

[60] McTavish N, Copeland L, Saville M, Perkins N, Spruce B (2007). Proenkephalin assists stress-activated apoptosis through transcriptional repression of NF-κB-and p53-regulated gene targets. Cell death & differentiation.

[61] Xiao F-Y, Nheu L, Komesaroff P, Ling S (2015). Testosterone protects cardiac myocytes from superoxide injury via NF-κB signalling pathways. Life sciences.

[62] Qiao L, Zhang H, Yu J, Francisco R, Dent P, Ebert MP (2006). Constitutive activation of NF-κB in human hepatocellular carcinoma: evidence of a cytoprotective role. Human gene therapy.

[63] Liemburg-Apers DC, Willems PH, Koopman WJ, Grefte S (2015). Interactions between mitochondrial reactive oxygen species and cellular glucose metabolism. Archives of toxicology.

[64] Robertson RP (2004). Chronic oxidative stress as a central mechanism for glucose toxicity in pancreatic islet beta cells in diabetes. Journal of Biological Chemistry.

[65] El-Osta A, Brasacchio D, Yao D, Pocai A, Jones PL, Roeder RG (2008). Transient high glucose causes persistent epigenetic changes and altered gene expression during subsequent normoglycemia. Journal of Experimental Medicine.

[66] Okabe J, Orlowski C, Balcerczyk A, Tikellis C, Thomas MC, Cooper ME (2012). Distinguishing Hyperglycemic Changes by Set7 in Vascular Endothelial CellsNovelty and Significance. Circulation research.

[67] Li Y, Reddy MA, Miao F, Shanmugam N, Yee J-K, Hawkins D (2008). Role of the histone H3 lysine 4 methyltransferase, SET7/9, in the regulation of NF-κB-dependent inflammatory genes relevance to diabetes and inflammation. Journal of Biological Chemistry.

[68] Yang XD, Huang B, Li M, Lamb A, Kelleher NL, Chen LF (2009). Negative regulation of NF-κB action by Set9-mediated lysine methylation of the RelA subunit. The EMBO journal.

[69] Mittenberg AG, Moiseeva TN, Barlev NA (2008). Role of proteasomes in transcription and their regulation by covalent modifications. Frontiers in Bioscience-Landmark.

[70] Zhang X, Tang N, Hadden TJ, Rishi AK (2011). Akt, FoxO and regulation of apoptosis. Biochimica et Biophysica Acta (BBA)-Molecular Cell Research.

[71] Fu Z, Tindall D (2008). FOXOs, cancer and regulation of apoptosis. Oncogene.

[72] Thompson MG, Larson M, Vidrine A, Barrios K, Navarro F, Meyers K (2015). FOXO3-NF-κB RelA protein complexes reduce proinflammatory cell signaling and function. The Journal of Immunology.

[73] Beretta GL, Corno C, Zaffaroni N, Perego P (2019). Role of FoxO proteins in cellular response to antitumor agents. Cancers.

[74] Fitzwalter BE, Thorburn A (2018). FOXO3 links autophagy to apoptosis. Autophagy.

[75] Eijkelenboom A, Burgering BM (2013). FOXOs: signalling integrators for homeostasis maintenance. Nature reviews Molecular cell biology.

[76] Lehtinen MK, Yuan Z, Boag PR, Yang Y, Villén J, Becker EB (2006). A conserved MST-FOXO signaling pathway mediates oxidative-stress responses and extends life span. Cell.

[77] Neri C (2012). Role and therapeutic potential of the pro-longevity factor FOXO and its regulators in neurodegenerative disease. Frontiers in pharmacology.

[78] Wang X, Hu S, Liu L (2017). Phosphorylation and acetylation modifications of FOXO3a: Independently or synergistically?. Oncology letters.

[79] Yang J-Y, Zong CS, Xia W, Yamaguchi H, Ding Q, Xie X (2008). ERK promotes tumorigenesis by inhibiting FOXO3a via MDM2-mediated degradation. Nature cell biology.

[80] Brunet A, Bonni A, Zigmond MJ, Lin MZ, Juo P, Hu LS (1999). Akt promotes cell survival by phosphorylating and inhibiting a Forkhead transcription factor. cell.

[81] Storz P (2011). Forkhead homeobox type O transcription factors in the responses to oxidative stress. Antioxidants & redox signaling.

[82] Olmos Y, Brosens JJ, Lam EW-F (2011). Interplay between SIRT proteins and tumour suppressor transcription factors in chemotherapeutic resistance of cancer. Drug Resistance Updates.

[83] Daitoku H, Sakamaki J-i, Fukamizu A (2011). Regulation of FoxO transcription factors by acetylation and protein-protein interactions. Biochimica et Biophysica Acta (BBA)-Molecular Cell Research.

[84] Hauck L, Harms C, Grothe D, An J, Gertz K, Kronenberg G (2007). Critical role for FoxO3a-dependent regulation of p21CIP1/WAF1 in response to statin signaling in cardiac myocytes. Circulation research.

[85] Carter ME, Brunet A (2007). FOXO transcription factors. Current Biology.

[86] Zrelli H, Matsuoka M, Kitazaki S, Zarrouk M, Miyazaki H (2011). Hydroxytyrosol reduces intracellular reactive oxygen species levels in vascular endothelial cells by upregulating catalase expression through the AMPK-FOXO3a pathway. European Journal of Pharmacology.

[87] Tan W-Q, Wang K, Lv D-Y, Li P-F (2008). Foxo3a inhibits cardiomyocyte hypertrophy through transactivating catalase. Journal of Biological Chemistry.

[88] Xie Q, Hao Y, Tao L, Peng S, Rao C, Chen H (2012). Lysine methylation of FOXO3 regulates oxidative stress-induced neuronal cell death. EMBO reports.

[89] Brunet A, Sweeney LB, Sturgill JF, Chua KF, Greer PL, Lin Y (2004). Stress-dependent regulation of FOXO transcription factors by the SIRT1 deacetylase. science.

[90] Tseng AH-H, Wu L-H, Shieh S-S, Wang DL (2014). SIRT3 interactions with FOXO3 acetylation, phosphorylation and ubiquitinylation mediate endothelial cell responses to hypoxia. Biochemical Journal.

[91] Oppenheimer H, Kumar A, Meir H, Schwartz I, Zini A, Haze A (2014). Set7/9 impacts COL2A1 expression through binding and repression of SirT1 histone deacetylation. Journal of Bone and Mineral Research.

[92] Xie Q, Peng S, Tao L, Ruan H, Yang Y, Li T-M (2014). E2F transcription factor 1 regulates cellular and organismal senescence by inhibiting Forkhead box O transcription factors. Journal of Biological Chemistry.

[93] Lezina L, Aksenova V, Ivanova T, Purmessur N, Antonov A, Tentler D (2014). KMTase Set7/9 is a critical regulator of E2F1 activity upon genotoxic stress. Cell death and differentiation.

[94] Gu Y, Wang X, Liu H, Li G, Yu W, Ma Q (2018). SET7/9 promotes hepatocellular carcinoma progression through regulation of E2F1. Oncology reports.

[95] He S, Owen DR, Jelinsky SA, Lin L-L (2015). Lysine methyltransferase SETD7 (SET7/9) regulates ROS signaling through mitochondria and NFE2L2/ARE pathway. Scientific reports.

[96] Rius-Pérez S, Torres-Cuevas I, Millán I, Ortega ÁL, Pérez S (2020). PGC-1α, inflammation, and oxidative stress: an integrative view in metabolism. Oxidative medicine and cellular longevity.

[97] Dang Y, Ma X, Li Y, Hao Q, Xie Y, Zhang Q (2018). Inhibition of SETD7 protects cardiomyocytes against hypoxia/reoxygenation-induced injury through regulating Keap1/Nrf2 signaling. Biomedicine & Pharmacotherapy.

[98] Wang C, Shu L, Zhang C, Li W, Wu R, Guo Y (2018). Histone methyltransferase Setd7 regulates Nrf2 signaling pathway by phenethyl isothiocyanate and ursolic acid in human prostate cancer cells. Molecular nutrition & food research.

[99] Smith KA, Waypa GB, Schumacker PT (2017). Redox signaling during hypoxia in mammalian cells. Redox biology.

[100] Hong S-S, Lee H, Kim K-W (2004). HIF-1alpha: a valid therapeutic target for tumor therapy. Cancer Res Treat.

[101] Cole RM, Hirsch AT, Henry TD (2016). Gene Therapy in Critical Limb Ischemia. Stem Cell and Gene Therapy for Cardiovascular Disease: Elsevier.

[102] Smith TG, Robbins PA, Ratcliffe PJ (2008). The human side of hypoxia-inducible factor. British journal of haematology.

[103] Schiliro C, Firestein BL (2021). Mechanisms of metabolic reprogramming in cancer cells supporting enhanced growth and proliferation. Cells.

[104] Kulichkova VA, Fedorova OA, Tsimokha AS, Moiseeva TN, Bottril A, Lezina L (2010). 26S proteasome exhibits endoribonuclease activity controlled by extra-cellular stimuli. Cell cycle.

[105] Kim Y, Nam HJ, Lee J, Kim C, Yu YS, Kim D (2016). Methylation-dependent regulation of HIF-1α stability restricts retinal and tumour angiogenesis. Nature Communications.

[106] Liu X, Wang D, Zhao Y, Tu B, Zheng Z, Wang L (2011). Methyltransferase Set7/9 regulates p53 activity by interacting with Sirtuin 1 (SIRT1). Proceedings of the National Academy of Sciences.

[107] Daks A, Shuvalov O, Fedorova O, Petukhov A, Lezina L, Zharova A p53-Independent Effects of Set7/9 Lysine Methyltransferase on Metabolism of Non-Small Cell Lung Cancer Cells. Frontiers in Oncology. 2021: 3985.

[108] Vafa O, Wade M, Kern S, Beeche M, Pandita TK, Hampton GM (2002). c-Myc can induce DNA damage, increase reactive oxygen species, and mitigate p53 function: a mechanism for oncogene-induced genetic instability. Molecular cell.

[109] Muthalagu N, Murphy DJ (2018). Is oxidative stress MYC's Achilles heel?. Cell Death and Differentiation.

[110] Yu L, Hitchler MJ, Sun W, Sarsour EH, Goswami PC, Klingelhutz AJ (2009). AP-2α inhibits c-MYC induced oxidative stress and apoptosis in HaCaT human keratinocytes. Journal of oncology.

[111] Sabnis HS, Somasagara RR, Bunting KD (2017). Targeting MYC dependence by metabolic inhibitors in cancer. Genes.

[112] Han X, Ren C, Lu C, Qiao P, Yang T, Yu Z (2022). Deubiquitination of MYC by OTUB1 contributes to HK2 mediated glycolysis and breast tumorigenesis. Cell Death & Differentiation.

[113] Hung C-L, Wang L-Y, Yu Y-L, Chen H-W, Srivastava S, Petrovics G (2014). A long noncoding RNA connects c-Myc to tumor metabolism. Proceedings of the National Academy of Sciences.

[114] Osthus RC, Shim H, Kim S, Li Q, Reddy R, Mukherjee M (2000). Deregulation of glucose transporter 1 and glycolytic gene expression by c-Myc. Journal of Biological Chemistry.

[115] Shim H, Dolde C, Lewis BC, Wu C-S, Dang G, Jungmann RA (1997). c-Myc transactivation of LDH-A: implications for tumor metabolism and growth. Proceedings of the national academy of sciences.

[116] Benassi B, Fanciulli M, Fiorentino F, Porrello A, Chiorino G, Loda M (2006). c-Myc phosphorylation is required for cellular response to oxidative stress. Molecular cell.

[117] Swindall AF, Stanley JA, Yang ES (2013). PARP-1: friend or foe of DNA damage and repair in tumorigenesis?. Cancers.

[118] Hsu P-C, Gopinath RK, Hsueh Y-A, Shieh S-Y (2019). CHK2-mediated regulation of PARP1 in oxidative DNA damage response. Oncogene.

[119] Du Y, Yamaguchi H, Wei Y, Hsu JL, Wang H-L, Hsu Y-H (2016). Blocking c-Met-mediated PARP1 phosphorylation enhances anti-tumor effects of PARP inhibitors. Nature medicine.

[120] Martín-Guerrero SM, Casado P, Hijazi M, Rajeeve V, Plaza-Díaz J, Abadía-Molina F (2020). PARP-1 activation after oxidative insult promotes energy stress-dependent phosphorylation of YAP1 and reduces cell viability. Biochemical Journal.

[121] Zhang S, Lin Y, Kim Y, Hande M, Liu Z, Shen H (2007). c-Jun N-terminal kinase mediates hydrogen peroxide-induced cell death via sustained poly (ADP-ribose) polymerase-1 activation. Cell Death & Differentiation.

[122] Ye N, Zhang N, Zhang Y, Qian H, Wu B, Sun Y (2019). Cul4a as a new interaction protein of PARP1 inhibits oxidative stress-induced H9c2 cell apoptosis. Oxidative Medicine and Cellular Longevity.

[123] Yeh T-YJ, Sbodio JI, Nguyen M, Meyer TN, Lee RM, Chi N-W (2005). Tankyrase-1 overexpression reduces genotoxin-induced cell death by inhibiting PARP1. Molecular and cellular biochemistry.

[124] Joshi A, Iyengar R, Joo J, Li-Harms X, Wright C, Marino R (2016). Nuclear ULK1 promotes cell death in response to oxidative stress through PARP1. Cell Death & Differentiation.

[125] Galluzzi L, Vitale I, Aaronson SA, Abrams JM, Adam D, Agostinis P (2018). Molecular mechanisms of cell death: recommendations of the Nomenclature Committee on Cell Death 2018. Cell Death & Differentiation.

[126] Mathews MT, Berk BC (2008). PARP-1 inhibition prevents oxidative and nitrosative stress-induced endothelial cell death via transactivation of the VEGF receptor 2. Arteriosclerosis, thrombosis, and vascular biology.

[127] Meng Y, Wu C, Yu B, Li H, Chen M, Qi G (2018). PARP-1 involvement in autophagy and their roles in apoptosis of vascular smooth muscle cells under oxidative stress. Folia Biol (Praha).

[128] Funato Y, Michiue T, Asashima M, Miki H (2006). The thioredoxin-related redox-regulating protein nucleoredoxin inhibits Wnt-β-catenin signalling through dishevelled. Nature cell biology.

[129] Kajla S, Mondol AS, Nagasawa A, Zhang Y, Kato M, Matsuno K (2012). A crucial role for Nox 1 in redox-dependent regulation of Wnt-β-catenin signaling. The FASEB Journal.

[130] Chatterjee S, Sil PC (2022). ROS-Influenced Regulatory Cross-Talk With Wnt Signaling Pathway During Perinatal Development. Frontiers in Molecular Biosciences.

[131] Tonelli C, Chio IIC, Tuveson DA (2018). Transcriptional regulation by Nrf2. Antioxidants & redox signaling.

[132] Shen C, Wang D, Liu X, Gu B, Du Y, Wei FZ (2015). SET7/9 regulates cancer cell proliferation by influencing β-catenin stability. The FASEB Journal.

[133] Althubiti M, Rada M, Samuel J, Escorsa JM, Najeeb H, Lee K-G (2016). BTK Modulates p53 Activity to Enhance Apoptotic and Senescent ResponsesRole of BTK in the p53 Pathway. Cancer research.

[134] Barlev NA, Liu L, Chehab NH, Mansfield K, Harris KG, Halazonetis TD (2001). Acetylation of p53 activates transcription through recruitment of coactivators/histone acetyltransferases. Molecular cell.

[135] Daks A, Melino D, Barlev N (2013). The role of different E3 ubiquitin ligases in regulation of the P53 tumor suppressor protein. Tsitologiia.

[136] Fischer M (2017). Census and evaluation of p53 target genes. Oncogene.

[137] Li Y, Jenkins CW, Nichols MA, Xiong Y (1994). Cell cycle expression and p53 regulation of the cyclin-dependent kinase inhibitor p21. Oncogene.

[138] Fridman JS, Lowe SW (2003). Control of apoptosis by p53. Oncogene.

[139] Fedorova O, Petukhov A, Daks A, Shuvalov O, Leonova T, Vasileva E (2019). Orphan receptor NR4A3 is a novel target of p53 that contributes to apoptosis. Oncogene.

[140] Fedorova O, Daks A, Petrova V, Petukhov A, Lezina L, Shuvalov O (2018). Novel isatin-derived molecules activate p53 via interference with Mdm2 to promote apoptosis. Cell Cycle.

[141] Liu D, Xu Y (2011). p53, oxidative stress, and aging. Antioxidants & redox signaling.

[142] Itahana K, Pervaiz S (2014). Crosstalk Between p53 and Mitochondrial Metabolism. Mitochondria: The Anti-Cancer Target for the Third Millennium: Springer.

[143] Hussain SP, Amstad P, He P, Robles A, Lupold S, Kaneko I (2004). p53-induced up-regulation of MnSOD and GPx but not catalase increases oxidative stress and apoptosis. Cancer research.

[144] Ding B, Chi SG, Kim SH, Kang S, Cho JH, Kim DS (2007). Role of p53 in antioxidant defense of HPV-positive cervical carcinoma cells following H2O2 exposure. Journal of cell science.

[145] Montero J, Dutta C, Van Bodegom D, Weinstock D, Letai A (2013). p53 regulates a non-apoptotic death induced by ROS. Cell Death & Differentiation.

[146] Huang J, Perez-Burgos L, Placek BJ, Sengupta R, Richter M, Dorsey JA (2006). Repression of p53 activity by Smyd2-mediated methylation. Nature.

[147] Tang Y, Luo J, Zhang W, Gu W (2006). Tip60-dependent acetylation of p53 modulates the decision between cell-cycle arrest and apoptosis. Molecular cell.

[148] Vaziri H, Dessain SK, Eaton EN, Imai S-I, Frye RA, Pandita TK (2001). hSIR2SIRT1 functions as an NAD-dependent p53 deacetylase. Cell.

[149] Espadinha M, Lopes EA, Marques V, Amaral JD, Dos Santos DJ, Mori M (2022). Discovery of MDM2-p53 and MDM4-p53 protein-protein interactions small molecule dual inhibitors. European Journal of Medicinal Chemistry.

[150] Bulatov E, Sayarova R, Mingaleeva R, Miftakhova R, Gomzikova M, Ignatyev Y (2018). Isatin-Schiff base-copper (II) complex induces cell death in p53-positive tumors. Cell Death Discovery.

[151] Lezina L, Aksenova V, Fedorova O, Malikova D, Shuvalov O, Antonov AV (2015). KMT Set7/9 affects genotoxic stress response via the Mdm2 axis. Oncotarget.

[152] Joshi S, Singh AR, Durden DL (2014). MDM2 regulates hypoxic hypoxia-inducible factor 1α stability in an E3 ligase, proteasome, and PTEN-phosphatidylinositol 3-kinase-AKT-dependent manner. Journal of Biological Chemistry.

[153] Yang W, Dolloff NG, El-Deiry WS (2008). ERK and MDM2 prey on FOXO3a. nature cell biology.

[154] Thomasova D, Mulay SR, Bruns H, Anders H-J (2012). p53-independent roles of MDM2 in NF-κB signaling: implications for cancer therapy, wound healing, and autoimmune diseases. Neoplasia.

[155] Kurash JK, Lei H, Shen Q, Marston WL, Granda BW, Fan H (2008). Methylation of p53 by Set7/9 mediates p53 acetylation and activity in vivo. Molecular cell.

[156] Tao Y, Neppl RL, Huang Z-P, Chen J, Tang R-H, Cao R (2011). The histone methyltransferase Set7/9 promotes myoblast differentiation and myofibril assembly. Journal of Cell Biology.

[157] Kim J-D, Kim E, Koun S, Ham H-J, Rhee M, Kim M-J (2015). Proper activity of histone H3 lysine 4 (H3K4) methyltransferase is required for morphogenesis during zebrafish cardiogenesis. Molecules and cells.

[158] Kofent J, Zhang J, Spagnoli FM (2016). The histone methyltransferase Setd7 promotes pancreatic progenitor identity. Development.

[159] Volpe CMO, Villar-Delfino PH, Dos Anjos PMF, Nogueira-Machado JA (2018). Cellular death, reactive oxygen species (ROS) and diabetic complications. Cell death & disease.

[160] Cencioni C, Spallotta F, Martelli F, Valente S, Mai A, Zeiher AM (2013). Oxidative stress and epigenetic regulation in ageing and age-related diseases. International journal of molecular sciences.

[161] Kwa FA, Thrimawithana TR (2014). Epigenetic modifications as potential therapeutic targets in age-related macular degeneration and diabetic retinopathy. Drug Discovery Today.

[162] Ogihara T, Vanderford NL, Maier B, Stein RW, Mirmira RG (2009). Expression and function of Set7/9 in pancreatic islets. Islets.

[163] Deering TG, Ogihara T, Trace AP, Maier B, Mirmira RG (2009). Methyltransferase Set7/9 maintains transcription and euchromatin structure at islet-enriched genes. Diabetes.

[164] Fujimaki K, Ogihara T, Morris DL, Oda H, Iida H, Fujitani Y (2015). SET7/9 enzyme regulates cytokine-induced expression of inducible nitric-oxide synthase through methylation of lysine 4 at histone 3 in the islet β cell. Journal of Biological Chemistry.

[165] Jetton TL, Flores-Bringas P, Leahy JL, Gupta D (2021). SetD7 (Set7/9) is a novel target of PPARγ that promotes the adaptive pancreatic β-cell glycemic response. Journal of Biological Chemistry.

[166] Maganti AV, Maier B, Tersey SA, Sampley ML, Mosley AL, Özcan S (2015). Transcriptional activity of the islet β cell factor Pdx1 is augmented by lysine methylation catalyzed by the methyltransferase Set7/9. Journal of Biological Chemistry.

[167] Chakrabarti SK, Francis J, Ziesmann SM, Garmey JC, Mirmira RG (2003). Covalent histone modifications underlie the developmental regulation of insulin gene transcription in pancreatic β cells. Journal of Biological Chemistry.

[168] Chen J, Guo Y, Zeng W, Huang L, Pang Q, Nie L (2014). ER stress triggers MCP-1 expression through SET7/9-induced histone methylation in the kidneys of db/db mice. American Journal of Physiology-Renal Physiology.

[169] Sharma N, Sankrityayan H, Kale A, Gaikwad AB (2020). Role of SET7/9 in the progression of ischemic renal injury in diabetic and non-diabetic rats. Biochemical and Biophysical Research Communications.

[170] Sasaki K, Doi S, Nakashima A, Irifuku T, Yamada K, Kokoroishi K (2016). Inhibition of SET domain-containing lysine methyltransferase 7/9 ameliorates renal fibrosis. Journal of the American society of nephrology.

[171] Frati G, Benedetto U, Biondi-Zoccai G, Sciarretta S (2015). Bridging the gap between translational and outcome research in cardiovascular disease. BioMed Research International.

[172] Raedschelders K, Ansley DM, Chen DD (2012). The cellular and molecular origin of reactive oxygen species generation during myocardial ischemia and reperfusion. Pharmacology & therapeutics.

[173] Ambrosini S, Montecucco F, Kolijn D, Pedicino D, Akhmedov A, Mohammed SA (2022). Methylation of the Hippo effector YAP by the methyltransferase SETD7 drives myocardial ischaemic injury: a translational study. Cardiovascular research.

[174] Yu C, Zhuang S (2019). Histone methyltransferases as therapeutic targets for kidney diseases. Frontiers in pharmacology.

[175] Chistiakov DA, Orekhov AN, Bobryshev YV (2017). Treatment of cardiovascular pathology with epigenetically active agents: Focus on natural and synthetic inhibitors of DNA methylation and histone deacetylation. International Journal of Cardiology.

[176] Kaniskan HUm, Martini ML, Jin J (2018). Inhibitors of protein methyltransferases and demethylases. Chemical reviews.

[177] Rugo HS, Jacobs I, Sharma S, Scappaticci F, Paul TA, Jensen-Pergakes K (2020). The promise for histone methyltransferase inhibitors for epigenetic therapy in clinical oncology: a narrative review. Advances in therapy.

[178] Hu C, Liu X, Zeng Y, Liu J, Wu F (2021). DNA methyltransferase inhibitors combination therapy for the treatment of solid tumor: mechanism and clinical application. Clinical Epigenetics.

[179] Barsyte-Lovejoy D, Li F, Oudhoff MJ, Tatlock JH, Dong A, Zeng H (2014). (R)-PFI-2 is a potent and selective inhibitor of SETD7 methyltransferase activity in cells. Proceedings of the National Academy of Sciences.

[180] Daks A, Mamontova V, Fedorova O, Petukhov A, Shuvalov O, Parfenyev S (2021). Set7/9 controls proliferation and genotoxic drug resistance of NSCLC cells. Biochemical and Biophysical Research Communications.

[181] Francis N-J, Rowlands M, Workman P, Jones K, Aherne W (2012). Small-molecule inhibitors of the protein methyltransferase SET7/9 identified in a high-throughput screen. Journal of biomolecular screening.

